# Noble Metals Doping in Lead‐Free Double Perovskite Single Crystals: Achieving Near‐Infrared to X‐ray Broadband Photodetection

**DOI:** 10.1002/smsc.202500135

**Published:** 2025-06-23

**Authors:** Donato Valli, Roel Vanden Brande, Vincent Herreman, Qianrui Li, Giacomo Romolini, Jim Jui‐Kai Chen, Muhammed Shameem K. M., Bob Van Hout, Li Sun, Qing Zhao, Bapi Pradhan, Johan Hofkens, Elke Debroye

**Affiliations:** ^1^ Department of Chemistry KU Leuven Celestijnenlaan 200F Heverlee 3001 Belgium; ^2^ Department of Chemistry University of Copenhagen Universitetsparken 5 DK‐2100 Copenhagen Denmark; ^3^ Cixi Institute of Biomedical Engineering Ningbo Institute of Materials Technology and Engineering Chinese Academy of Sciences Ningbo 315201 P. R. China; ^4^ School of Physics Peking University Beijing 100871 China

**Keywords:** Cs_2_AgBiBr_6_, doping, lead‐free perovskites, near-infrared, photodetectors, X‐rays

## Abstract

Semiconductor materials capable of broadband photodetection, spanning X‐rays to near‐infrared (NIR), are essential for applications in medical imaging, industrial inspection, security, and telecommunications. Conventional photodetectors like Si, Ge, InGaAs, and amorphous Se (a‐Se) often encounter tradeoffs in efficiency or cost‐effectiveness. Halide perovskites (HPs) offer competitive or superior optoelectronic properties with low‐cost, solution‐based processing. However, lead‐based HPs pose toxicity and stability challenges, while lead‐free tin‐based HPs suffer from Sn^2+^ oxidation and structural degradation. The lead‐free double perovskite Cs_2_AgBiBr_6_ has emerged as a stable, nontoxic alternative for X‐ray and visible‐light photodetection. Despite its advantages, its high bandgap (≈1.9 eV) limits NIR absorption. This study explores doping Cs_2_AgBiBr_6_ with noble metal cations (Au^3+^, Pd^2+^, and Ir^3+^) to lower its absorption onset and enhance its photodetection capabilities across a broad spectrum. The results demonstrate that noble metal doping can overcome the intrinsic limitations of pristine Cs_2_AgBiBr_6_, enabling efficient photodetection from X‐rays to the NIR range. This approach highlights a viable pathway for developing next‐generation broadband photodetectors that combine nontoxicity, stability, and wide‐spectrum sensitivity.

## Graphical abstract

This study examines the doping of Cs_2_AgBiBr_6_ double perovskite with noble metals (Au^3+^, Pd^2+^, and Ir^3+^). Significant redshifts in the absorption edge enable broadband photodetection from X‐rays to NIR, without abrupt changes in crystal structure. Au doping notably also enhances X‐ray sensitivity. This doping approach offers a straightforward strategy for broadband photodetection with potentials in the wider field of optoelectronics.

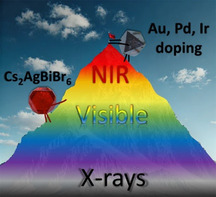

## 1 Introduction

The radiation detection of X‐ray to NIR wavelengths spans numerous application fields, including medical imaging, industrial inspection, security, and telecommunication, just to name a few.^[^ ^1^, ^2^, ^3^, ^4^, ^5^ ^]^ Commonly used broad‐range photodetectors, such as those based on Si, Ge, InGaAs, or amorphous Se (a‐Se) offer a robust performance but suffer from several drawbacks.^[^ ^2^ ^]^ Si photodetectors, for instance, exhibit limited sensitivity in the NIR region due to their bandgap energy and a low efficiency for X‐ray detection due to their low atomic number, while InGaAs requires cooling during operation due to its high dark current and risk of degradation.^[^ ^3^, ^4^, ^6^ ^]^ Additionally, they require high‐temperature processing and precise control over doping levels, which results in high production costs.^[^ ^1^, ^2^ ^]^ In recent years, halide perovskite semiconductors have emerged as promising alternatives for a vast range of optoelectronic applications, including photodetection.^[^ ^5^, ^7^, ^8^, ^9^, ^10^, ^11^, ^12^ ^]^ These materials, particularly lead‐based perovskites, have demonstrated excellent optoelectronic properties, such as high absorption coefficients, interesting charge‐carrier dynamics, and a high degree of structural and photophysical tunability.^[^ ^13^ ^]^ However, the toxicity of lead and their relative instability toward environmental factors remain significant concerns, shifting considerable research efforts to lead‐free alternatives.^[^ ^9^ ^]^ One promising candidate is the double perovskite Cs_2_AgBiBr_6_, offering a nontoxic and highly stable alternative while maintaining many beneficial properties of its lead‐based counterparts.^[^ ^14^, ^15^ ^]^ Cs_2_AgBiBr_6_ has already demonstrated significant potential in various applications, especially in the field of X‐ray photodetection reaching sensitivities up to thousands of μC Gy^−1^ cm^−2^.^[^ ^9^, ^14^, ^16^, ^17^ ^]^ However, its relatively wide bandgap, ≈1.90 eV, limits its absorption range to energies in the visible range, below the orange/red part of the electromagnetic spectrum. To address this drawback, doping has been proven to be a good strategy to lower the bandgap of Cs_2_AgBiBr_6_ and at the same time enhancing the optoelectronic performance.^[^ ^17^, ^18^, ^19^ ^]^ Incorporation of dopants such as Tl, Sn, Cu, Fe, Ru, and Pd into the crystal structure has resulted in redshifting the absorption edge toward the NIR up to 1400 nm, even allowing for photodetection in this range.^[^ ^20^, ^21^, ^22^, ^23^, ^24^, ^25^, ^26^ ^]^ Building on similar prior advances in doping strategies and NIR photodetection, this study is the first to demonstrate broad‐range photodetection—from X‐rays to the NIR—using noble‐metal‐doped Cs_2_AgBiBr_6_ single crystals.

Specifically, Au^3+^, Pd^2+^, and Ir^3+^ were selected for their demonstrated ability to form perovskite(‐like) materials, their comparable ionic radii with Ag^+^ and Bi^3+^, and the stability of their oxidation states.^[^ ^27^, ^28^, ^29^ ^]^ Each dopant also possesses partially filled d‐orbitals—5d^8^ (Au^3+^), 5d^6^ (Ir^3+^), and 4d^8^ (Pd^2+^)—that can effectively modify the Cs_2_AgBiBr_6_ lattice by creating additional energy states near the band edges, thus narrowing the bandgap and extending absorption into the NIR.^[^ ^23^, ^24^, ^25^, ^26^, ^27^, ^28^ ^]^ Because Au^3+^ and Ir^3+^ both exhibit a + 3 oxidation state, unlike Pd^2+^, the exact origin of the NIR absorption can differ between these dopants.^[^ ^23^, ^24^, ^25^, ^26^ ^]^ As proof of fact, high‐quality doped single crystals exhibited a significant redshift in their absorption edge, extending into the NIR region, reaching up to 1100 and 1600 nm for Au‐, Ir‐, and Pd‐doping respectively, without altering the structural characteristics of the pristine composition. Photodetection tests showed that the Au‐doped crystals, in particular, exhibited promising performance across the NIR, visible, and X‐ray regions, with a fourfold increase in X‐ray sensitivity compared to pristine Cs_2_AgBiBr_6_. These findings suggest that noble metal cations doping offers a promising approach to extend the absorption range of pristine Cs_2_AgBiBr_6_, enabling broad‐range photodetection while retaining the enhanced stability and ease of synthesis of lead‐free double perovskite. Eventually, this approach highlights a viable pathway for developing next‐generation broadband photodetectors that combine nontoxicity, stability, and wide‐spectrum sensitivity.

## 2 Results and Discussion

**Figure 1:**
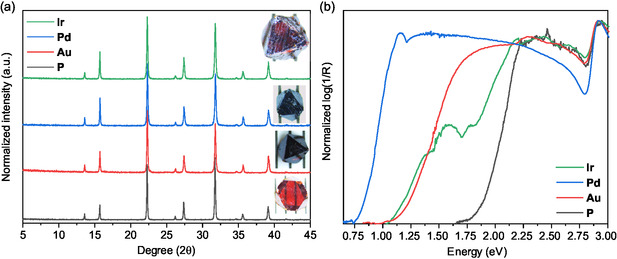
a) PXRD patterns of the ground single crystals (pictures as insets) showing a consistent crystal structure for the **P** (black) and the **Au** (red), **Pd** (blue), and **Ir** (green) single crystals. b) Log(1/R) conversion of the diffuse reflectance spectra confirms the redshift of the bandgap, corresponding to the observed color change in the single crystals.

The Cs_2_AgBiBr_6_ pristine (**P**) and doped single crystals (SCs) were synthesized using a modified slow‐cooling method from a supersaturated solution,^[^ ^16^ ^]^ specifically adjusted to allow to incorporate noble metal cations as dopants (Au^3+^, Pd^2+^, and Ir^3+^). Two distinct parameters were evaluated and compared to the synthesis protocol of the pristine material, specifically the temperature cooling rate and the dopant concentration (Figure S1, Supporting Information). Similar effects were found for slow cooling and a higher concentration of the dopants on the one hand and faster cooling and low concentration of the dopants on the other hand. Based on those observations, we have determined a 1% stoichiometric ratio compared to Bi as the optimal doping level for maintaining high‐quality Au‐ and Pd‐doped single crystals grown at a cooling rate of 1.5–2 °C per hour (Figure S1b, Supporting Information). Higher doping levels or slower cooling rates (Figure S1c, Supporting Information) resulted in the formation of single crystals with high surface roughness and consisting of uncontrolled mixed phases with impurities possibly from reprecipitation of the precursor material. On the other hand, a cooling profile faster than 1.5 °C per hour resulted in the separate precipitation of the pristine material and the reagent or the formation of small crystal aggregates due to the formation of multiple nucleation sites within the solution (Figure S1d, Supporting Information). It must be noted that, for Ir doping, higher stoichiometric ratios up to 5% were used since no trace of Ir was present in the crystal with lower Ir content. 5% Ir doping led to both the formation of Ir‐poor and Ir‐rich phases as evident from dark red and black zones, respectively, within the same single crystal (Figure S1b, Supporting Information). All synthesized SCs exhibited a darker color depending on the dopant, as previously observed for Cu, Fe, and Ru doping.^[^ ^23^, ^24^, ^25^ ^]^ Au‐ and Pd‐doped SCs were black colored, while Ir‐doped SCs were dark red with visible black spots. Given the expected low effective doping concentration in the SCs, inductively coupled plasma optical emission spectroscopy (ICP‐OES) was first employed to precisely define the elemental composition (Table S1, Supporting Information). Despite that the qualitative presence of the dopants was demonstrated, an exact composition could not be obtained via this technique due to the inability to completely dissolve the materials. As an alternative, a study via X‐ray fluorescence (XRF) also confirmed the qualitative presence of the dopants, rather than providing their exact concentration (Table S2, Supporting Information). The different (un)doped materials will be referred to from now on as **P** for the pristine Cs_2_AgBiBr_6_ composition, **Au** for Cs_2_AgBiBr_6_:Au, **Pd** for Cs_2_AgBiBr_6_:Pd, and **Ir** for Cs_2_AgBiBr_6_:Ir. Powder X‐ray diffraction analysis (PXRD) (**Figure** **1**a) and Rietveld refinement did not reveal any significant differences in the crystal structure across all samples, as expected upon employing low doping amounts, regardless of the doping site (Figure S2, Supporting Information).^[^ ^18^, ^22^, ^30^ ^]^ The refinement confirmed the retention of the cubic structure (Fm$\bar{3}$m) with only minor changes in the lattice parameters, which are ascribed to the smaller ionic radii of the dopants as compared to Bi^3+^ and or Ag^+^ (Table S3, Supporting Information).^[^ ^14^, ^29^ ^]^ These changes were barely visible as a shift in the diffraction peaks to slightly higher two theta values for the doped material, hence lattice contraction, when compared to the pristine material. Moreover, the PXRD patterns revealed no peak‐splitting or the formation of additional ones, suggesting the absence of side phases, such as Cs_2_Au_2_Br_6_ (3D structure) or the Cs_2_AgPdBr_5_‐like phase (1D structure).^[^ ^27^, ^31^, ^32^ ^]^ This also confirms the successful phase‐pure synthesis, as it is well‐known that Cs_3_Bi_2_Br_9_ might form alongside Cs_2_AgBiBr_6_. Focusing on **Ir** samples, they were characterized by a red color with black spots inside the SCs (Figure 1a, inset). However, from its PXRD pattern, the black regions possess the same phase as **P** (absence of extra peaks), but are probably more dopant‐rich (black color) as opposed to the rest of the material.

In line with the PXRD findings, Raman spectroscopy also showed consistent spectra across all samples (Figure S3, Supporting Information). The characteristic peaks originating from AgBr_6_ and BiBr_6_ octahedra vibrations centered around 70 cm^−1^ (T_2g_ Ag‐Br scissoring), 131 cm^−1^ (E_2g_ Bi‐Br asymmetric stretching) and 174 cm^−1^ (A_1g_ symmetric Ag and Bi‐Br symmetric stretching) were the same for the pristine and doped samples.^[^ ^33^ ^]^ An exception was found for the E_2g_ stretching mode of **Pd**, which was positioned at 134 cm^−1^ with a width of 34 cm^−1^ as opposed to the 16 cm^−1^ found for the other materials. This difference can be explained by the lattice contraction caused by Pd doping in the pristine material and the disorder induced by the charge mismatch at either the Ag^+^ or Bi^3+^ site. Anyway, the similarity among the spectra indicates that the doping evenly affects the surface and bulk of the crystal rather than resulting in dopants precipitation on the double perovskite material surface. During the slow‐cooling step of the synthesis, the different solubility of the Ir‐salt as compared to the other reagents might have led to the formation of small Ir‐rich Cs_2_AgBiBr_6_ clusters—small black particles formed in solution prior to SCs growth—acting as nucleation centers, around which the Ir‐poor structure is then formed.

Next, it is anticipated that doping Cs_2_AgBiBr_6_ SCs with noble metal cations primarily affects the optical properties of the material, rather than causing profound structural modifications. This observation is in line with previous reports.^[^ ^22^, ^23^, ^24^, ^25^, ^26^ ^]^ To this note, the diffuse reflectance spectra in Figure 1b show a shift of the absorption edge of the materials toward the NIR upon doping, correlating with the observed change in color of the single crystals. Relative to the pristine material with an edge at 660 nm (1.88 eV), Pd doping resulted in the largest shift, ≈1600 nm (0.78 eV), followed by Ir and Au doping to about 1100 nm (1.13 eV). The spectral line shapes varied among the doped samples. The shift found for **Pd** even surpasses the 1400 nm absorption onset previously reported by Lei et al. for Pd doping in Cs_2_AgBiBr_6_.^[^ ^26^ ^]^ **Au** and **Pd** showed a uniform shift of the absorption onset to lower energies. On the other hand, **Ir** demonstrated a step‐like behavior with both a stronger and smaller, though noticeable, redshift of the entire absorption onset. This particular appearance of the absorption onset for **Ir** could be linked to the presence of both Ir‐rich (absorption from 1.13 to 1.75 eV) and Ir‐poor (from 1.75 eV onward) phases (Figure S4, Supporting Information). The precise origin of this NIR transition for the three dopants can be attributed to two different mechanisms. The first one, as reported by Zhang et al. depends on the ability of the Bi‐site dopant, in their case Ru^3+^, to form an intermediate band (IB) ascribable to the Ru d‐orbitals positioned in the forbidden gap of the pristine material.^[^ ^24^ ^]^ These electronic energy levels, positioned below the Fermi level, will allow eventually for additional excitation pathways from the IB to the conduction band (CB). Furthermore, they also reported that the resulting IB is relatively flat, indicating a small curvature, which is linked to a high photo‐carrier effective mass, hence small mobility. This implies that the transport properties of the doped Cs_2_AgBiBr_6_ will change, more specifically being lowered upon Ru‐doping. The second process/mechanism, found for Cu^+/2+^ doping, was reported by Ji et al.^[^ ^23^ ^]^ In their study, they proposed that the NIR shift of the absorption edge arises from the formation of deep‐level defects, rather than a direct contribution of the dopant to the band structure itself. In this case, the photogeneration of carriers can occur both via the band‐to‐band and via defect‐to‐band transitions, as also confirmed by Lei et al. in the case of Pd doping.^[^ ^26^ ^]^ It should be noted that the photoconductivity for this type of transition in semiconductors is usually small for sub‐bandgap excitation compared to band‐to‐band excitation due to the typically low density of defect states and low optical cross section for defect‐to‐band absorption. Based on the reported mechanisms, it is straightforward to assign the origin for the NIR transition in the **Au** sample to the first process, while due to the charge mismatch of Pd^2+^ with either Ag^+^ or Bi^3+^, the second mechanism would be more plausible. However, a more in‐depth discussion on the possible mechanism follows after testing the radiation detection performance across different wavelengths.

**Figure 2:**
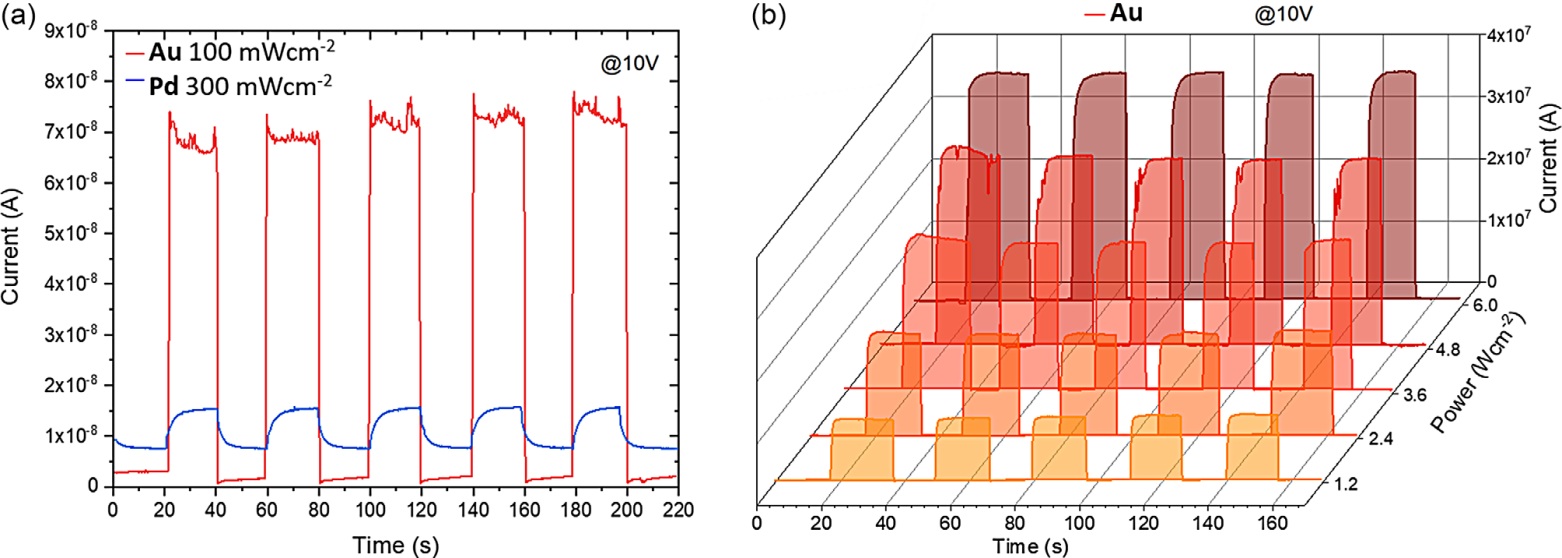
a) IT characteristics for **Au** and **Pd** under 785 nm excitation and b) IT characteristics for **Au** under 1064 nm excitation upon increasing the laser excitation power, showing a positive photoresponse under NIR excitation. A 10 V bias was applied in both cases.

Given the evident redshift of the absorption onset, the materials’ potential photoresponse in a broad range of excitation spanning from the NIR‐I and NIR‐II regions (785 and 1064 nm, respectively), over the visible, up to the X‐ray region was investigated. Due to the nonuniformity of the **Ir** sample and the resulting poor photoresponse, the following discussion on the photodetection capabilities of the doped materials will focus on **Au** and **Pd**, while the optoelectronic data for **Ir** are provided in the supplementary information (Figure S5, Supporting Information). Prior to recording the current‐time (IT) characteristics at 10 V, parallel electrode devices (Au/perovskite/Au) were fabricated by thermally evaporating Au contacts on two opposite faces of the single crystals. 785 nm laser illumination served as starting point for the broad‐range photodetection tests, with the pristine material device being used as control sample. As expected, no photocurrent was recorded for **P**, whose bandgap lies in the visible region of the electromagnetic spectrum (Figure S6, Supporting Information). In contrast, both **Au** and **Pd** exhibited a photoresponse in the nA range, with resulting on/off ratios of 55 and 2 (**Figure** **2**a) under 100 and 300 mW cm^−2^, respectively. Note that an increase in power density was necessary to achieve an on/off ratio of 2 for **Pd** (Figure S7, Supporting Information). The photocurrent was found to be relatively stable under different on/off cycles. Moreover, these values were found to be in line with the ones reported for Ru doping (2,980 nm, 85 mW cm^−2^) and Fe doping (50,808 nm, 115 mW cm^−2^).^[^ ^19^, ^20^ ^]^ To compare the devices’ performance within the broader field of lead‐free perovskite materials, the values of responsivity (R), and specific detectivity (D*) were calculated for the best‐performing **Au** device, according to the following Equation (1) and (2).^[^ ^1^ ^]^

(1) $$R=\frac{I_{\text{ph}}-I_{d}}{P\times A}$$

(2) $$D^{*}=\frac{R\sqrt{A}}{\sqrt{2eI_{d}}}$$

In these formulas, *I*_ph_ is defined as the photocurrent, *I*_d_ as the dark current, *P* as the excitation power, *A* as the photoactive surface area, and *e* as the elementary charge. The obtained values were 64.8 mA W^−1^ and 10^8^ Jones, respectively. These performances are lower than those of Si photodetectors (*R* = 0.6–0.7 A W^−1^ and D* ≈ 10^12^ Jones), InGaAs photodetectors (*R* = 0.9–1.0 AW^−1^ and D* ≈ 10^13^ Jones), and the best‐performing Sn‐based HP devices (*R* = 0.5 A W^−1^ and D* ≈ 10^12^ Jones) (Table S4, Supporting Information).^[^ ^5^, ^34^, ^35^ ^]^ Despite the lower values of **Au** device, they represent a promising starting point to extend the photodetection capabilities of halide double perovskite‐based down to the NIR, overcoming its limitation to the visible range.^[^ ^2^, ^9^, ^26^ ^]^ Additionally, the rise (τ_r_) and decay times (τ_d_), defined as the time needed to reach 90% of the total photocurrent from 10% and vice versa, are in the order of milliseconds (τ_r_ = 468 ms and τ_d_ = 250 ms) being also in line with other lead‐free perovskite materials.^[^ ^9^ ^]^ Nevertheless, the material stability and ease of synthesis are superior compared to the other materials. It is known that Sn‐based HPs require precise control over the (post‐)synthesis to avoid the oxidation of Sn^2+^ to Sn^4+^, Si and InGaAs‐based detectors rely on costly high‐temperature vacuum deposition methods, and specifically for InGaAs, cooling during operation due to its high dark‐current and risk of degradation is mandatory.^[^ ^3^, ^6^ ^]^ Attracted by the positive photoresponse under 785 nm excitation, the samples were also tested under high‐intensity (1.3–6.2 W cm^−2^) 1064 nm laser excitation to mimic the conditions used in NIR imaging. As shown in Figure 2b and Figure S8, Supporting Information, both materials exhibit an increase in photocurrent in the tens of nA to μA range, with on/off ratios nonlinearly and linearly increasing with increasing laser intensity for **Pd** and **Au**, respectively. The photocurrent is higher than under 785 nm excitation due to the higher laser power, while still stable over time. However, the devices’ response slowed down to the second range. The overall responsivity is lower than that under 785 nm excitation, which is a common phenomenon arising when the photodetecting material is subjected to a drastic increase in illumination power due to the loss in photodetecting gain.^[^ ^36^ ^]^ On top of that, it must be considered that the lower responsivity for **Au** might arise from the laser excitation energy being close to the band edge (lower absorption cross section), while not being the case for the **Pd** sample.

All in all, while the performance of the **Au** and **Pd** samples is below that of InGaAs, the current market golden standard, it should be noted that, particularly for **Pd**, its extended absorption addresses a key drawback of Si photodetectors, their low sensitivity below/above 1100 nm/1.13 eV. This opens interesting avenues for material improvements. For instance, by combining Au^3+^ and Pd^2+^ doping, it may be possible to retain the optoelectronic properties of **Au** while still extending the absorption range as in **Pd** to 1600 nm, potentially reaching the quality of InGaAs, which is commonly used up to 1700 nm (although its responsivity slowly decreases at longer wavelengths to 0.6–0.8 A W^−1^). Moreover, the ability to mildly modify the electronic structure of Cs_2_AgBiBr_6_ double perovskite to potentially operate up to 1600 nm is groundbreaking for all perovskite‐based material, which are typically limited by a bandgap that only reaches the red part of the spectrum at best in its standard composition.

**Figure 3:**
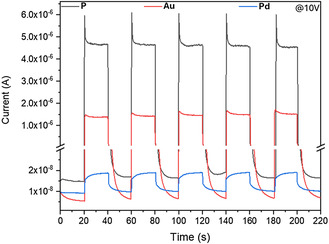
IT characteristics for **P** (black), Au‐doped (red), and Pd‐doped (blue) samples under AM1.5 conditions, showing a positive photoresponse under a 10 V bias.

Following the study of the NIR photodetection properties of the doped materials, their broad‐range photodetection capabilities were further tested with visible light photodetection. In this frame, solar irradiation (AM1.5) was generated by a Xe lamp at a power of 100 mW cm^−2^. As depicted in **Figure** **3**, the synthesized materials show an increase in current up to the range of tens of μA when exposed to light under 10 V bias, while **Au** also shows the lowest dark current among the three materials. On/off ratios of 274 and 223 were calculated for **P** and **Au**, respectively, while only being 2 for **Pd**.

Small differences in the photocurrent, hence the on/off ratio, might originate from the different areas of the samples exposed to light, with **P** (1.2 mm^2^) exposing the largest, followed by **Au** (1 mm^2^) and **Pd** (0.8 mm^2^). However, these differences are not enough to account for the differences between **P** and **Au** compared to **Pd**, as will be discussed later. In addition to this difference in photoresponse, **Pd** also is characterized by slower rise and decay times (*τ*_r_ = 6.13 s and *τ*_d_ = 5.86 s) compared to **P** and **Au** (*τ*_r_ = 192 and *τ*_d_ = 255 ms for **P**, and *τ*_r_ = 255 and *τ*_d_ = 461 ms for **Au**) (Figure S9, Supporting Information).

**Figure 4:**
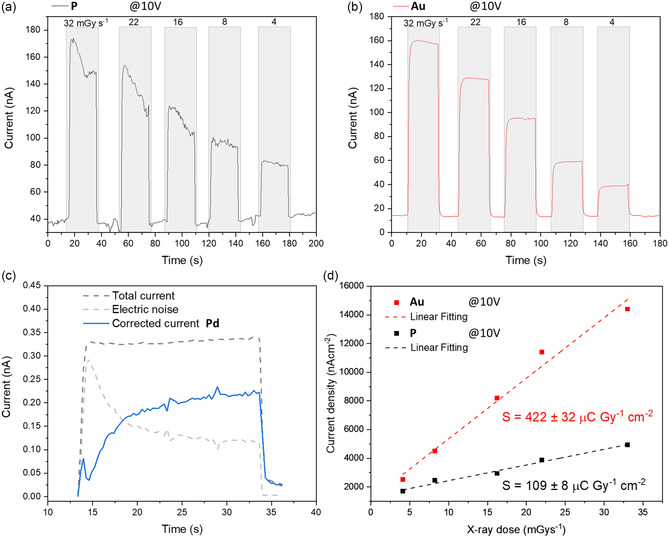
IT characteristics for a) **P** and b) **Au** under an X‐ray beam at different dose rates. c) IT characteristics under 32 mGy s^−1^ for **Pd**, showing the corrected signal, total current, and background noise. d) Calculated sensitivities for **P** and **Au**, showing a nearly four times increase. A 10 V bias was applied in all cases.

As a final step to evaluate the broadband photodetection capability of the doped materials, the photoresponse under X‐ray exposure was tested (**Figure** **4**). A tungsten target X‐ray tube with a varying dose rate (up to 32 mGys^−1^) was employed and the bias voltage was set to 10 V. To benchmark the different materials, the X‐ray sensitivity (S) value (Equation 3), was calculated (Figure 4d) with *D* corresponding to the X‐ray dose.^[^ ^37^ ^]^

(3) $$S=\frac{I_{\text{ph}}-I_{d}}{D\times A}$$

The S for the **P** (109 μC Gy_air_^−1^ cm^−2^) aligns with other reports on double perovskite materials positioning themselves in the 10–1000 range μC Gy_air_^−1^ cm^−2^ (Table S5, Supporting Information). However, it should be considered that a relatively lower electric field (10 V mm^−1^ on average) has been used as compared to reports showing a higher sensitivity value.^[^ ^37^ ^]^ Moving to **Au**, the *S* value is about 4 times higher (422 μC Gy_air_^−1^ cm^−2^).

Moreover, **Au** also shows a lower dark‐current consistent with the IT‐characteristics under white light. This interesting result points out that Au doping can be both used to expand the detection range of the pristine material and boost the X‐ray performance of the pristine composition. This increase in X‐ray performance is higher than the one previously reported for phenylethyl ammonium substitution and in line with the one reported for Rb doping (Cs‐site modifications).^[^ ^18^, ^22^ ^]^ Regarding **Pd**, even at the highest dose, the photocurrent was very low, in the order of hundreds of pA, and very close to the electrical noise from the instrumentation itself.^[^ ^1^, ^9^, ^37^, ^38^, ^39^, ^40^ ^]^

Based on the broad‐range photodetection study, it is evident that there is a clear trend among the doped materials, regardless of the excitation source. Specifically, **Au** demonstrates similar or superior performance as compared to **P**, with the added advantage of a lower dark current and expanded absorption range. **Au** outperforms **Pd** across all the tested ranges, despite the higher bandgap of **Au**, which will eventually limit its photodetection at wavelengths below 1100 nm. While **Pd** allows for broad‐range photodetection, its performance is relatively low. One reason for this different behavior might originate from the different ionic charges of the dopants. The d‐orbitals of Au^3+^ might contribute to additional electronic states in the forbidden gap of the pristine material, serving as intermediate‐to‐conduction band transitions, similar to Ru^3+^ doping.^[^ ^24^ ^]^ Pd^2+^, on the other hand, is likely to induce defect levels due to the charge mismatch with the Ag^+^ and Bi^3+^ cations.^[^ ^26^ ^]^ Given the close‐packed structure of double perovskite materials, it is most probable that Pd‐doping will result in Ag, Br, Bi vacancies (V_Ag,Br,Bi_), Ag‐Bi antisites (Ag_Bi_), or a combination of these, rather than interstitials.^[^ ^13^ ^]^ Among the mentioned point defects, V_Bi_ would act as a deep acceptor level defect, while Ag_Bi_ as a deep donor level, potentially allowing for the transitions in the NIR region. Clearly, the ability of the dopants to induce a large amount of defect types widens the defect landscape. Together with the different doping amounts inferred in our study, this might be the origin of the different absorption onset values found with respect to the previously reported Pd‐doped double perovskite material.^[^ ^26^ ^]^ Although these defects exhibit a relatively large absorption cross section comparable to the band‐to‐band transition (Figure 1b), they do not enable a high photodetection efficiency, as also observed in silicon doping where high‐density deep‐level defects form an additional band inside the bandgap.^[^ ^23^ ^]^ This limitation may be due to the relative concentration of the point defects and the resulting position of the Fermi level, which might trap a large number of photoexcited carriers, thus hampering the detection performance. This effect is particularly evident in the photoresponse of Pd‐doped material under different regimes, such as X‐ray and white‐light photodetection, as well as under relatively low‐fluence 785 nm excitation. In these cases, a noticeable decrease in performance is observed, along with a slower response time compared to the Au‐doped counterpart. Furthermore, Pd^2+^ has an electronic configuration of [Kr]4d,^8^ which leads to a preferential formation of square planar complexes.^[^ ^29^ ^]^ Hence, in a forced octahedral coordination, Pd^2+^ may loosely bind the axial Br^−−^ ions (Jahn–Teller‐like geometry), leading to a higher dark current due to ionic migration of the loosely bound axial halogen ions, which possess the lowest ion migration activation energy.^[^ ^41^ ^]^ On the other hand, this harmful phenomenon appears to be mitigated in the **Au** samples as shown by their lower dark current regardless of the excitation source as compared to the pristine material. Nevertheless, further scientific evidence is needed to clarify the exact effect of the noble metal cation dopants on the band structure, which could be explored through DFT calculations and studies on defect‐specific passivation. It should be noted that this NIR absorption does not compromise the well‐known Cs_2_AgBiBr_6_ high stability, which is paramount toward commercial applications (Figure S10, Supporting Information). The doped single crystals have been stored under ambient conditions for 6 months, and their PXRD patterns recorded afterward show no significant changes in their crystalline structure (Figure S11, Supporting Information).

## 3 Conclusions

The incorporation of minimal amounts of noble metal salts into Cs_2_AgBiBr_6_ double perovskite enabled photodetection spanning from X‐rays to the NIR region, thereby demonstrating for the first time the broad‐range potential of this doping strategy. Specifically, significant redshifts in the absorption edge were observed, resulting in absorption onsets at ≈1100 nm for **Au** and **Ir** and around 1600 nm for **Pd**. Structural analyses, including PXRD and Raman spectroscopy, confirmed the retention of the cubic structure with minimal changes in lattice parameters and the absence of side phases (at least for **Au** and **Pd**). More importantly, photodetection studies under various excitation sources, spanning from NIR, over the visible range, to X‐rays, demonstrated that **Au** crystals exhibited superior performance with a lower dark current as compared to **P** and **Pd**. The presence of two phases (Ir‐rich and Ir‐poor) in the **Ir** samples hampered its photoconducting properties. Interestingly, **Au** resulted in a fourfold increase in X‐ray sensitivity. These differences in behavior may originate from the distinct effects of the dopants on the band structure and defect landscape of the pristine material, providing a basis for further studies. In future experiments, the detection range could be extended to gamma rays. Moreover, this doping approach could be applied to various synthetic methods and halide perovskite compositions, similar to those already utilized in perovskite‐based photodetection studies, and could even be extended through the use of plasmonic nanoparticles as dopants, for example, in Au nanoparticle–perovskite hybrids.^[^ ^42^, ^43^, ^44^, ^45^ ^]^ All in all, these preliminary findings hold practical significance for NIR‐based health monitoring—particularly in applications such as oxygenation and hemodynamics—as well as for general X‐ray imaging. Additionally, the impact of this research extends beyond photodetection to solar cell technology, underscoring the potential of the transition metal doping strategy.^[^ ^45^ ^]^

## 4 Experimental Section

4.1

4.1.1

##### Material Synthesis

Cs_2_AgBiBr_6_ single crystals were grown following previously reported synthesis protocols and adjusting the temperature profile depending on the dopant solubilities (Figure S1, Supporting Information).^[^ ^16^ ^]^ A mixture containing 1.0 mmol of BiBr_3_ (≥98%, Sigma–Aldrich), 2.0 mmol of CsBr (99.9%, Sigma–Aldrich), 1.0 mmol of AgBr (≥99%, Chem‐Lab), and a 1 and 5% molar ratio compared to BiBr_3_ of AuBr_3_ (99.9%, Sigma–Aldrich) and PdBr_2_ (99.9%, Sigma–Aldrich) or IrCl_3_ **•**H_2_O (Sigma–Aldrich) were added in 10.5 mL of HBr (reagent grade, 48% w, Acros Organics). First, 10 min of sonication was performed, after which the mixture was heated to 120 °C for 3 h and the resulting clear solution was cooled to 40 °C over 3 days. The formed single crystals were washed with isopropanol (HPLC grade, Sigma–Aldrich) and vacuum dried at 60 °C for at least 6 h.

##### Elemental Analysis

ICP‐OES samples in the range of 1–10 ppm were prepared by digesting the corresponding ground SCs in concentrated HNO_3_ (70%) at 60 °C for 6 h. A white‐ish precipitate formed during the digestion step and could not be dissolved even by employing stronger digestion procedures (e.g., microwave, HF, H_2_SO_4_, and aqua regia). XRF analysis was conducted on bare SCs on a wavelength dispersive X‐ray fluorescence (WD‐XRF) Bruker S8 TIGER 4 K, equipped with a Rh anode. The measurements were performed under atmospheric helium with the samples placed in polyethylene cups (XRF Scientific) with a 4 μm prolene film support (Chemplex). It should be considered that the amount of sample used for the analysis (>1 g) did not reach the suggested minimum requirement.

##### Powder X‐ray Diffraction

PXRD patterns were recorded with a Malvern PANalytical Empyrean diffractometer. The instrument was set in transmission mode and equipped with a PIXcel3D solid‐state detector and a Cu anode X‐ray tube. The diffractograms, in the range of 5 to 45 2*θ* degrees, were refined through the FULLPROF program using Rietveld fitting with the same starting structural parameters as ref. [46, 47].

##### UV–Vis Diffuse Reflectance Spectroscopy

Diffuse‐reflectance spectroscopy spectra were recorded on a Lambda 950 UV−vis spectrophotometer (PerkinElmer) using BaSO_4_ powder and a beam blocker as a white and black reference respectively.

##### Device Fabrication

Single crystals with homogeneous surfaces close to 1 × 1 mm^[^ ^2^ ^]^ were chosen to fabricate photoconductor (Au/perovskite/Au) devices. 80 nm‐thick Au layers were deposited using a thermal evaporator.

##### NIR to X‐ray Photodetection

The noncoated surface of the devices was exposed first to two laser NIR excitation sources 785 nm (100 and 300 mW cm^−2^ for **Au** and **Pd** and **Ir** respectively) and 1064 nm (1.3–6.2 W cm^−2^, IPG Photonics). Following the NIR studies, a Xe lamp (Newport) was used to mimic the AM1.5 conditions (100 mW cm^−2^). Lastly, X‐ray photodetection was performed thanks to a commercial X‐ray imaging cabinet (XStrahl) equipped with a tungsten anode X‐ray tube allowing for precise X‐ray dose control. A Keithley 2400 source meter was used to control the bias voltage (kept constant at 10 V for all the ranges and materials) and record the resulting currents with and without incident photons. A dark environment was needed due to the high photodetecting capabilities of the materials.

## Conflict of Interest

The authors declare no conflict of interest.

## Author Contributions

**Donato Valli**: conceptualization (equal); data curation (equal); formal analysis (lead); funding acquisition (equal); investigation (equal); methodology (equal); writing—original draft (equal); writing—review editing (equal). **Roel Vanden Brande**: data curation (supporting); formal analysis (supporting); investigation (supporting). **Vincent Herreman**: data curation (supporting); formal analysis (supporting); investigation (supporting). **Qianrui Li**: data curation (supporting); formal analysis (supporting); investigation (supporting). **Giacomo Romolini**: data curation (supporting); formal analysis (supporting); investigation (supporting). **Jim Jui‐Kai Chen**: data curation (supporting); formal analysis (supporting); investigation (supporting). **Muhammed Shameem K. M.**: data curation (supporting); formal analysis (supporting); investigation (supporting). **Bob Van Hout**: data curation (supporting); formal analysis (supporting); investigation (supporting). **Li Sun**: data curation (supporting); investigation (supporting); writing—review editing (supporting). **Qing Zhao**: writing—review editing (supporting). **Bapi Pradhan**: data curation (supporting); investigation (supporting); writing—review editing (supporting). **Johan Hofkens**: conceptualization:equal; funding acquisition (equal); writing—original draft (supporting); writing—review editing (equal). **Elke Debroye**: conceptualization (equal); data curation (equal); formal analysis (equal); funding acquisition (lead); investigation (equal); supervision (lead); validation (equal); visualization (equal); writing—original draft (equal); writing—review editing (equal).

## Supporting information

Supplementary Material

## Data Availability

The data that support the findings of this study are available from the corresponding author upon reasonable request.
