# Supplementary material for: Noble Metals Doping in Lead‐Free Double Perovskite Single Crystals: Achieving Near‐Infrared to X‐ray Broadband Photodetection
Source: Small Sci. 2025 Jun 23;5(8):2500135. doi: 10.1002/smsc.202500135 (PMC12362788; doi:10.1002/smsc.202500135)
Supplement: Supplementary file 1 — Supplementary Material [file SMSC-5-2500135-s001.pdf]

Supporting Information

Noble Metals Doping in Lead-Free Double Perovskite Single Crystals: Achieving Near-Infrared to X-ray Broadband Photodetection

*Donato Valli, Roel Vanden Brande, Vincent Herreman, Qianrui Li, Giacomo Romolini, Jim Jui-Kai Chen, Muhammed Shameem K.M., Bob Van Hout, Li Sun, Qing Zhao, Bapi Pradhan, Johan Hofkens, Elke Debroye**

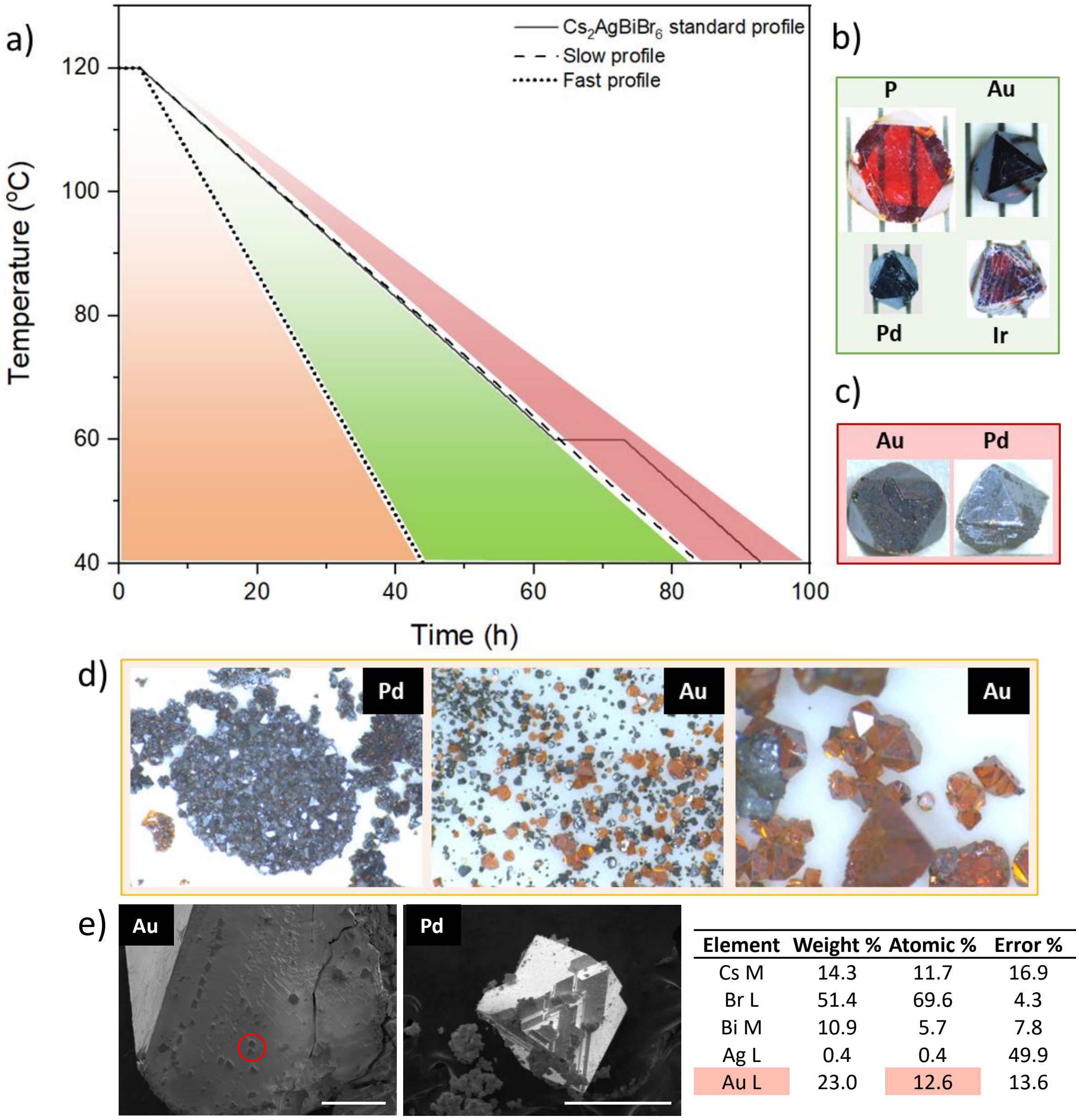

Element	Weight %	Atomic %	Error %
Cs M	14.3	11.7	16.9
Br L	51.4	69.6	4.3
Bi M	10.9	5.7	7.8
Ag L	0.4	0.4	49.9
Au L	23.0	12.6	13.6

Figure S1. (a) Temperature profile corresponding to the synthesis of (un)doped $Cs_2AgBiBr_6$ single crystals (SCs) (solid line). Three regions delimited by a dashed and dotted lines comprehend a faster region (orange) optimal region (green) and slower region (red) for the

growth of doped materials. b-d) The shaded boxes correspond to the resulting materials from each temperature-time zone. **P**, **Au**, **Pd**, and **Ir** refer to the pristine composition, and **Au-**, **Pd-**, and **Ir**-doped materials, respectively. b) The green zone resulted in the optimal single crystals in terms of dimensions, morphology, and composition. The smaller dimensions of the doped crystals compared to the pristine double perovskite may result from the slightly faster cooling rate used for the dopant-containing solution. This faster rate favors the formation of multiple nucleation sites, which, by growing independently, deplete the solution of reagents and thus hinder the further growth of the doped material.[1] Differences in the chemical potential of the various starting solutions may also be a contributing factor.[2] It should be noted that the Ir-doped single crystals are characterized by both black (Ir-rich) and dark-red (Ir-poor) zones. c) The slow cooling or higher dopant concentration (red zone) led to surface roughness and poor single-crystal morphology. d) Fast cooling or low dopant concentration (orange zone) led to the formation of small single crystals, which were unsuitable for further device studies, and reagents inclusions and reprecipitation. e) Representative SEM images showing the poor morphology and superficial reprecipitation of reagents on the **Au** (left) and **Pd** (right) 5% doped materials; the EDS report in the right panel corresponds to the area within the red circle.

Table S1. ICP-OES data for the **Au** (red) and **Pd** (blue) samples and resulting doping amounts. It should be noted that these data correspond to partially digested samples, hence they should be treated qualitatively. They mainly served to confirm the presence of dopants in the structure.

	**Au**	**Pd**
Bi (mg/L)	0.199167	0.149046
Au (mg/L)	0.000279	/
Pd (mg/L)	/	0.000853
Doping %	0.140	0.572

Table S2. XRF calculated moles, stoichiometries and resulting doping amounts compared to the amount of Bi for the Au- (red), Pd- (blue) and Ir- (green) doped samples. It should be considered that an over-estimation in the moles amount might have derived from the relatively little amount of sample available for the measurement.

	**Au**		**Pd**		**Ir**	
Atom	Moles	Stoichiometry	Moles	Stoichiometry	Moles	Stoichiometry
Br	0.535	6.000	0.536	6.000	0.531	6.000
Cs	0.263	2.951	0.263	2.944	0.263	2.972

Bi	0.058	0.649	0.063	0.701	0.063	0.708
Ag	0.070	0.782	0.081	0.903	0.075	0.843
Dopant	0.002	0.020 (3%)	0.001	0.009 (1%)	0.001	0.017 (2%)

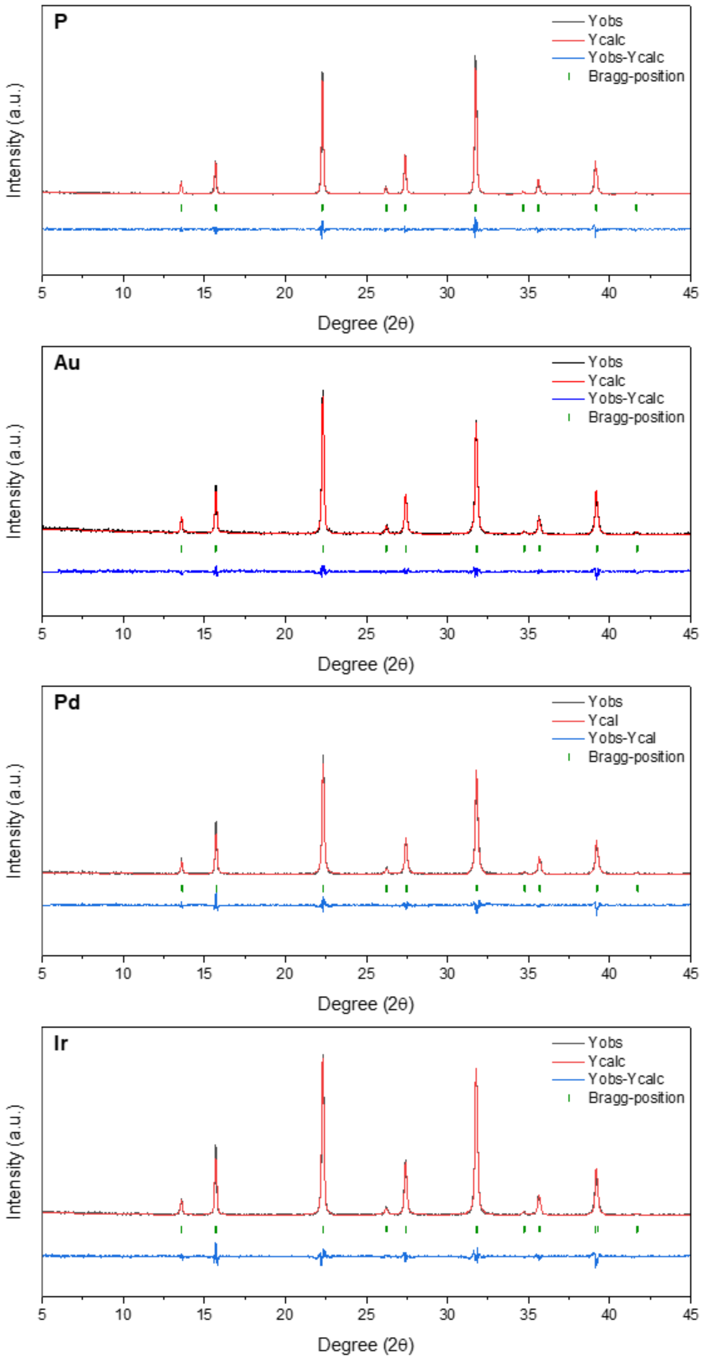

Figure S2. Rietveld refinements of the un(doped) (**P**, **Au**, **Pd**, **Ir**) perovskite ground SCs.

Table S3. Ionic radius of the substituted pristine and dopants cations considering a coordination number of 6, and corresponding lattice parameters for the pristine and doped samples.[3,4]

Material	Ionic radius (Å)	Lattice parameter (Å)
**P**	1.15 (Ag^{+})/1.03 (Bi^{3+})	11.2661
**Au**	0.99 (Au^{3+})	11.2550
**Pd**	1.00 (Pd^{2+})	11.2507
**Ir**	0.82 (Ir^{3+})	11.2544

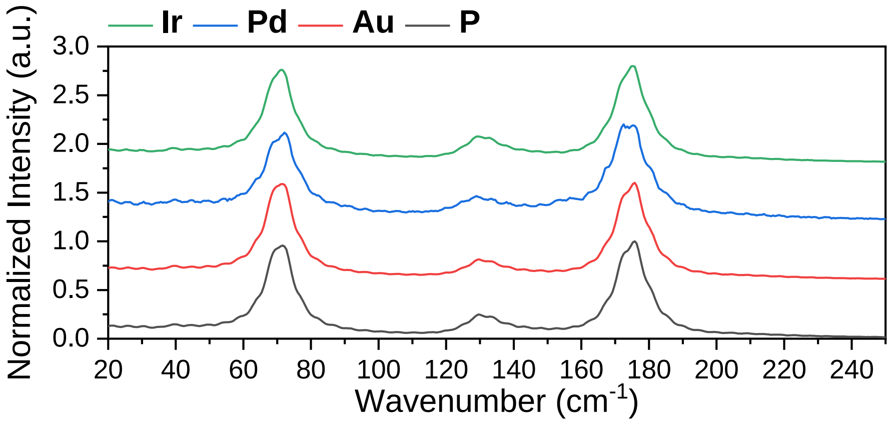

Figure S3. Raman spectra for the different samples, collected at a wavelength of 785 nm, confirming that doping did not induce any structural difference.

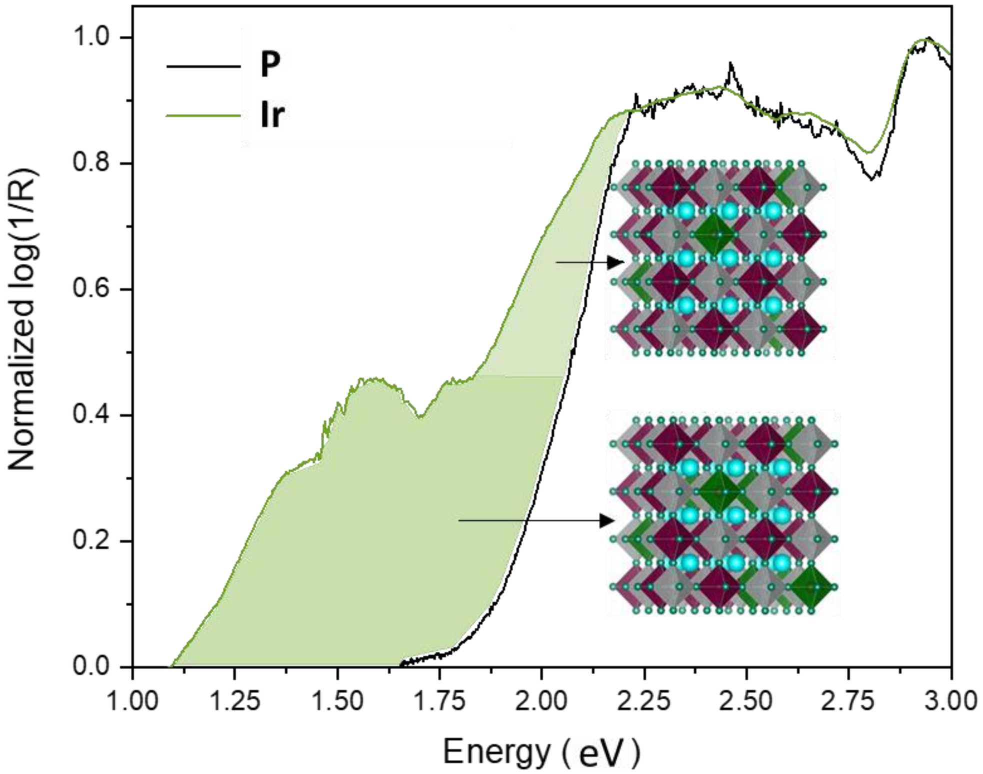

Figure S4. log(1/R) spectrum of the **Ir**-doped SCs representing the **Ir**-poor (light green) and **Ir**-rich (dark-green) absorption ranges.

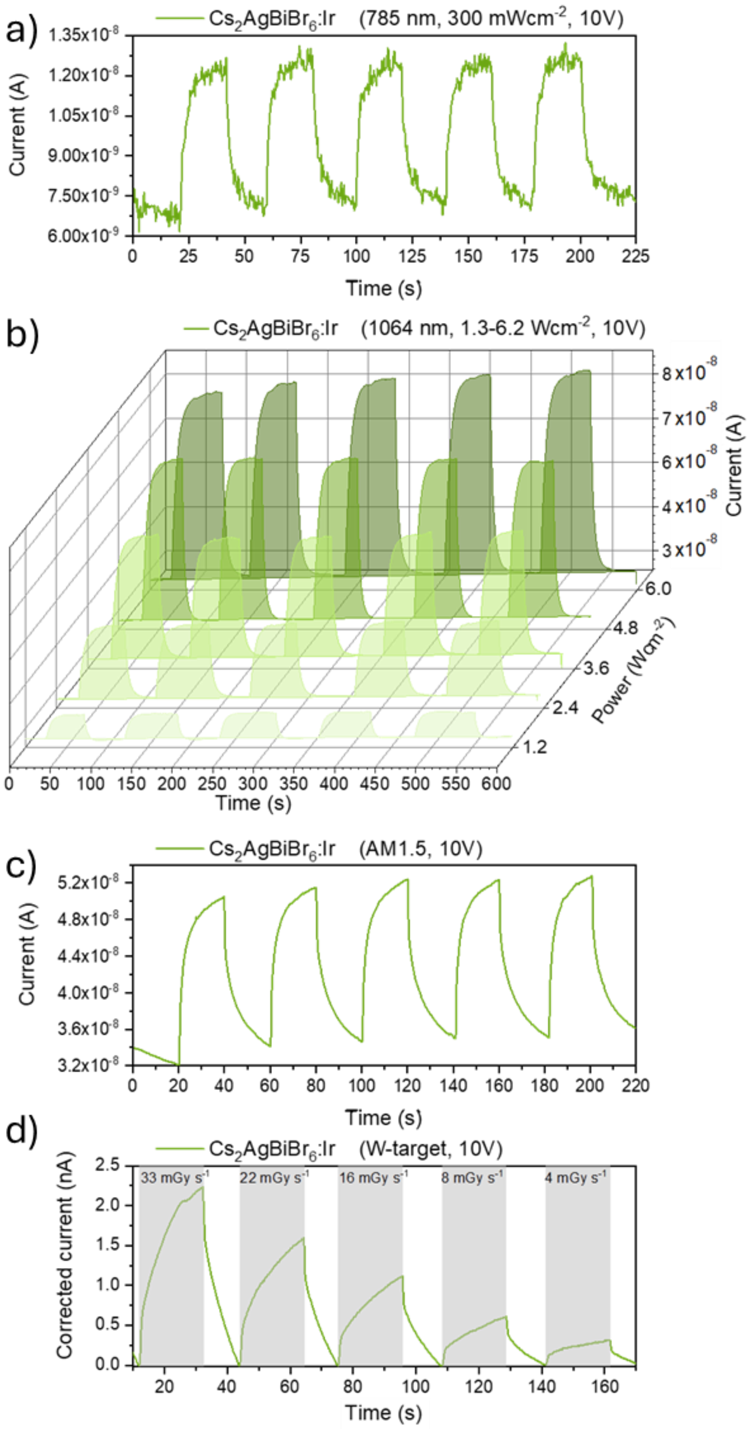

Figure S5. Ir current-time (IT) characteristics under near infrared (785 nm and 1064 nm, a,b respectively), AM1.5 (c) and X-ray (d) excitation.

The low photocurrent of the **Ir** samples can be ascribed to compositional inhomogeneities within the single crystal, namely the presence of Ir-rich and Ir-poor regions. This inhomogeneity gives rise to two primary effects. First, the boundaries between Ir-rich and Ir-poor zones can act as trap centers, quenching the photogenerated charge carriers as evident from the reduced white-light and X-ray photocurrent. Second, only the Ir-rich regions are capable of

absorbing infrared light; consequently, the overall crystal cannot absorb a sufficient amount of NIR radiation to generate a significant photocurrent in this region.

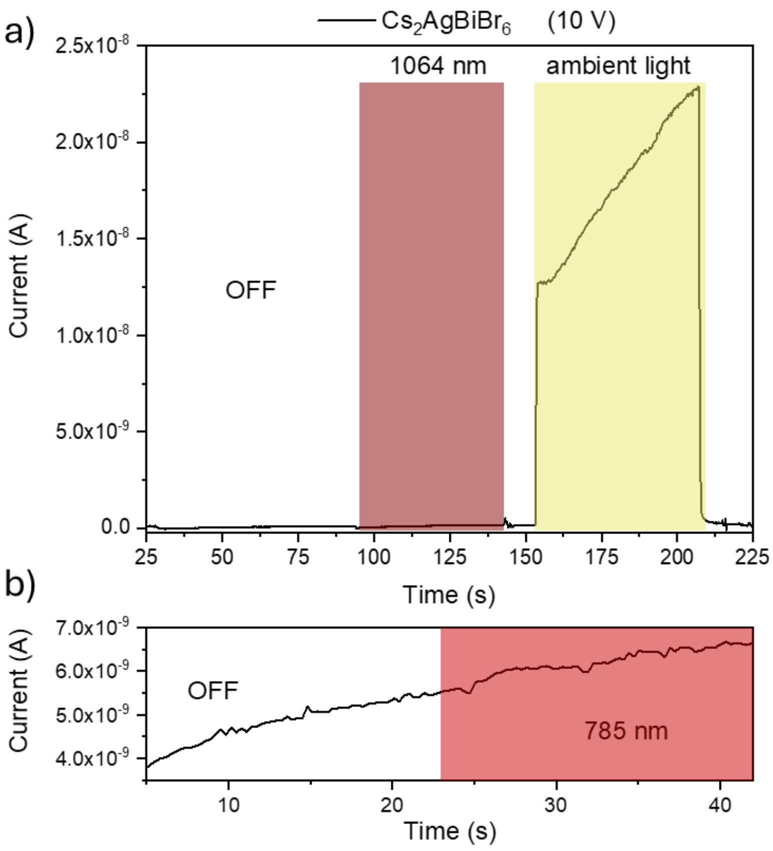

Figure S6. IT characteristics for **P** showing no photoresponse under near infrared excitation (785 nm, b; 1064 nm, a). The gradual rise in current upon switching on the light seen in (a) is attributed to the increasing irradiation intensity of the incandescent bulb used as ambient light source as it warms up.

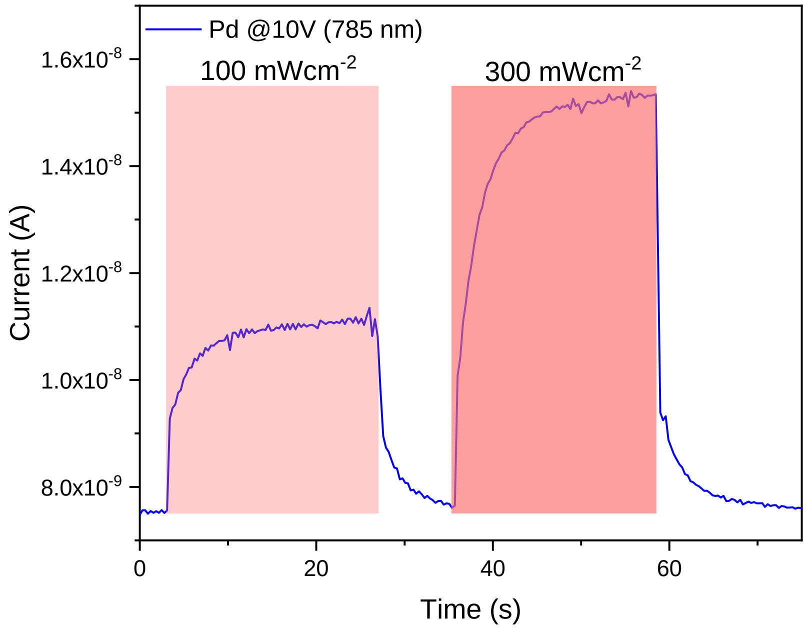

Figure S7. Photoresponse for the **Pd** based single crystal device at 10 V bias and 785 nm laser excitation under 100 and 300 mW cm^{-2} irradiation power.

Table S4. Figures of merit for different lead-free halide perovskites-based NIR photodetectors. For a broader comparison with Pb-based and hybrid perovskite-semiconductor photodetectors we refer to [5]. MA = methylammonium, FA = formamidinium, PEA = phenylethyl ammonium. [a] The value next to the dash corresponds to the dark-current range.

Material	Type of device and Conditions	ON/OFF ratio	Responsivity (mAW^{-1})	Specific Detectivity (Jones)	Rise/decay time (ms)	Ref.
**Au**	SC, 785 nm, 10V	55	65	1x10^{8}	468/250	This work
Si	400-1000 nm	/	500	3x10^{12}	/	[6]
$CsSnI_3$	Nanowire array, 940 nm, 0.1V	/ µA	54	3.85x10^{5}	83.8/243.4	[7]
$CsSnI_3$	Thin-film, 850 nm, 10 mV	/ µA	257	1.5x10^{11}	0.35/1.6	[8]
$MASnI_3$	Nanowire array, 300-1000 nm	/ nA	470	8.8x10^{10}	1.5/0.4 x10^{3}	[9]
$FASnI_3$	Thin film, 685 nm, 0.5V	/ µA	1.7x10^{8}	1.9x10^{12}	180/360 x10^{3}	[10]
$FASnI_3$	Thin film, 850 nm, 0.5V	/ µA	2x10^{8}	3x10^{12}	117/206 x10^{3}	[11]

$PEA_{0.15}FA_{0.85}SnI_3$	Thin film, 450-850 nm	/	390	8.29x10^{11}	0.78 x10^{-3}	[12]
$EDA_{0.01}FA_{0.98}SnI_3$:Ge	Thin film, 450-850 nm	/	340	1.61x10^{12}	34/54 x10^{-3}	[13]
$Cs_3Bi_2I_9$	Thin film, 450-950 nm	nA/pA	3.8	1.6x10^{12}	88.66/109.3	[14]
$MA_3Bi_2I_9$	Thin film, 400-700 nm	/	160	4.6x10^{12}	34.77/39.46	[15]

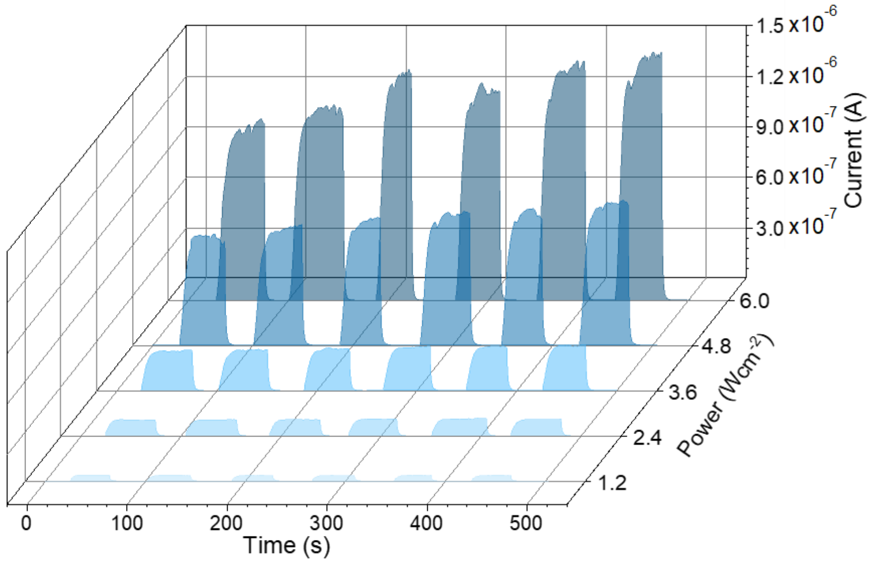

Figure S8. IT characteristics for the Pd-doped samples under 1064 nm excitation and 10 V bias showing a positive photoresponse under NIR excitation.

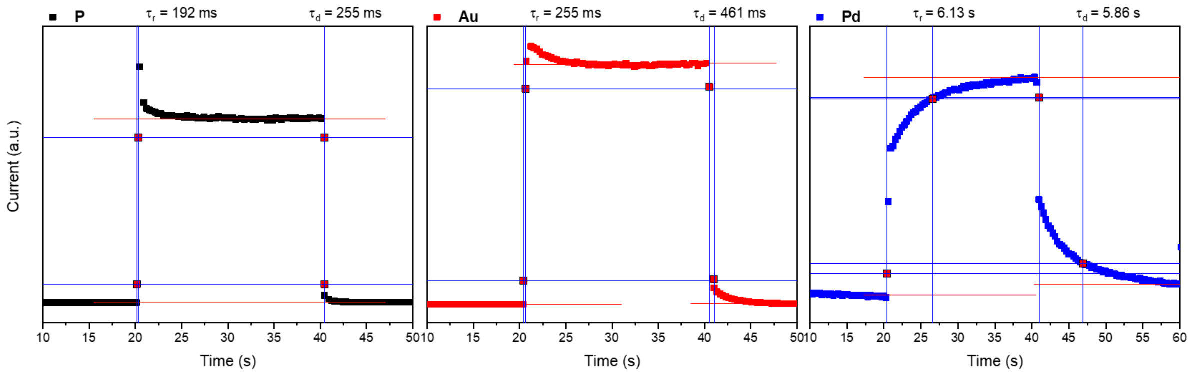

Figure S9. Rise (τ_r) and decay times (τ_d) for the **P**, **Au** and **Pd** samples at 10 V bias and under white light illumination (100 mW cm^{-2}).

Table S5. Sensitivities and corresponding electric field conditions for different double perovskite-based X-ray photodetectors. For a comparison within the broader field of lead-free perovskite materials we refer the reader to ref.[16]

Material	Electric field (V mm^{-1})	Sensitivity (μC Gy_{air}^{-1} cm^{-2})	Ref.
**P** SC	10	109	This work
**Au** SC	10	422	This work
a-Se	10000	20	[17]
$Cs_2AgBiBr_6$ SC	6	316	[18]
$Cs_2AgBiBr_6$ SC	25	105	[19]
$Cs_2AgBiBr_6$ SC	50	316-988 (298-77K)	[20]
$Cs_2AgBiBr_6$ wafer	1000	250	[21]
PEA-$Cs_2AgBiBr_6$ SC	22.7	288.8	[22]
$Cs_2AgBiCl_6$ SC	40	325.78	[23]

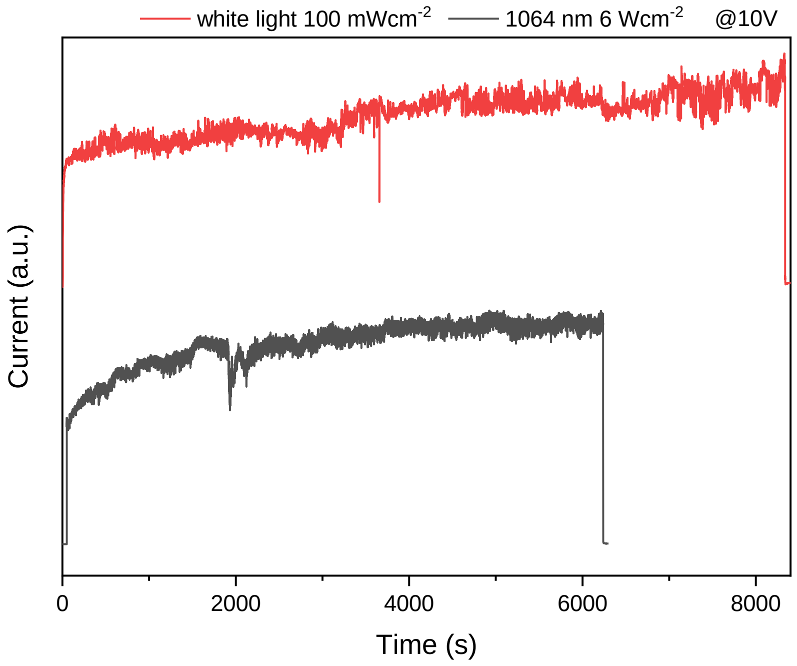

Figure S10. Long-term photoresponse stability of Au-doped $Cs_2AgBiBr_6$ under different illumination conditions. The black curve represents the photocurrent under 1064 nm illumination at an intensity of 6 W cm^{-2}, while the red curve corresponds to white light illumination at 100 mW cm^{-2}. The device was biased at 10 V, showing stable performance over time with no significant signs of degradation.

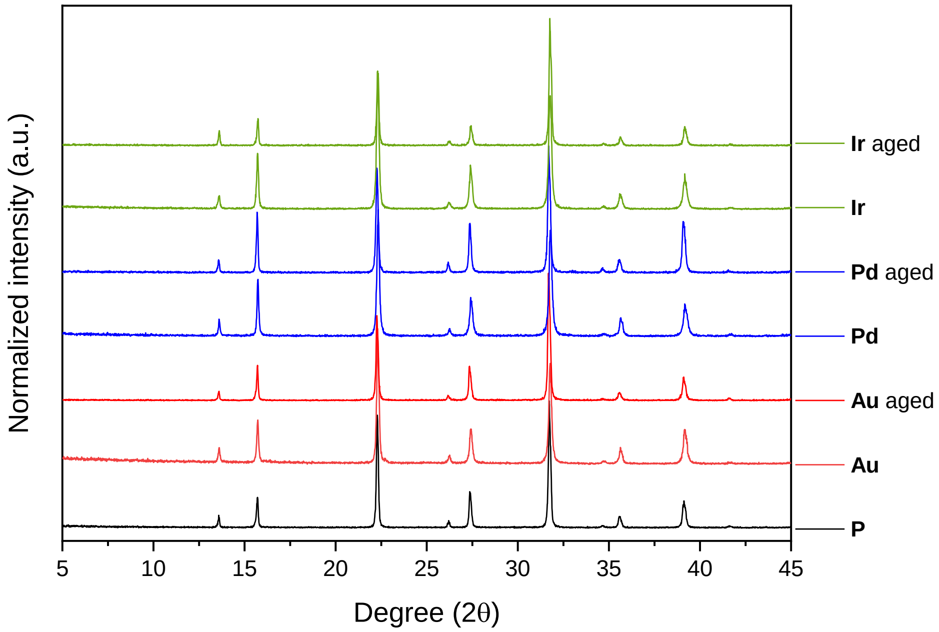

Figure S11. PXRD patterns of the pristine and doped materials, both freshly prepared and after six months of storage under ambient conditions, showing no significant changes in crystal structure.

Reference

(1) Yin, L.; Wu, H.; Pan, W.; Yang, B.; Li, P.; Luo, J.; Niu, G.; Tang, J. Controlled Cooling for Synthesis of Cs2AgBiBr6 Single Crystals and Its Application for X-Ray Detection. Adv. Opt. Mater. 2019, 7 (19), 1900491. https://doi.org/10.1002/adom.201900491.

(2) Yin, W.-J.; Shi, T.; Yan, Y. Unusual Defect Physics in CH3NH3PbI3 Perovskite Solar Cell Absorber. Appl. Phys. Lett. 2014, 104 (6), 063903. https://doi.org/10.1063/1.4864778.

(3) Lei, H.; Hardy, D.; Gao, F. Lead-Free Double Perovskite Cs2AgBiBr6: Fundamentals, Applications, and Perspectives. Adv. Funct. Mater. 2021, 31 (49), 2105898. https://doi.org/10.1002/adfm.202105898.

(4) Cotton, F. A.; Wilkinson, G.; Murillo, C. A.; Bochmann, A. Advanced Inorganic Chemistry, 6th Edition, 6th Edition.; Wiley-Interscience: New York, 1999.

(5) Gao, W.-H.; Chen, C. Perovskites and Their Constructed Near-Infrared Photodetectors. Nano Energy 2024, 128, 109904. https://doi.org/10.1016/j.nanoen.2024.109904.

(6) Saran, R.; Curry, R. J. Lead Sulphide Nanocrystal Photodetector Technologies. Nat. Photonics 2016, 10 (2), 81–92. https://doi.org/10.1038/nphoton.2015.280.

(7) Han, M.; Sun, J.; Peng, M.; Han, N.; Chen, Z.; Liu, D.; Guo, Y.; Zhao, S.; Shan, C.; Xu, T.; Hao, X.; Hu, W.; Yang, Z. Controllable Growth of Lead-Free All-Inorganic Perovskite Nanowire Array with Fast and Stable Near-Infrared Photodetection. J. Phys. Chem. C 2019, 123 (28), 17566–17573. https://doi.org/10.1021/acs.jpcc.9b03289.

(8) Cao, F.; Tian, W.; Wang, M.; Wang, M.; Li, L. Stability Enhancement of Lead-Free CsSnI3 Perovskite Photodetector with Reductive Ascorbic Acid Additive. InfoMat 2020, 2 (3), 577–584. https://doi.org/10.1002/inf2.12074.

(9) Waleed, A.; Tavakoli, M. M.; Gu, L.; Wang, Z.; Zhang, D.; Manikandan, A.; Zhang, Q.; Zhang, R.; Chueh, Y.-L.; Fan, Z. Lead-Free Perovskite Nanowire Array Photodetectors with Drastically Improved Stability in Nanoengineering Templates. Nano Lett. 2017, 17 (1), 523–530. https://doi.org/10.1021/acs.nanolett.6b04587.

(10) Liu, C.-K.; Tai, Q.; Wang, N.; Tang, G.; Loi, H.-L.; Yan, F. Sn-Based Perovskite for Highly Sensitive Photodetectors. Adv. Sci. 2019, 6 (17), 1900751. https://doi.org/10.1002/advs.201900751.

(11) Liu, C.-K.; Tai, Q.; Wang, N.; Tang, G.; Hu, Z.; Yan, F. Lead-Free Perovskite/Organic Semiconductor Vertical Heterojunction for Highly Sensitive Photodetectors. ACS Appl. Mater. Interfaces 2020, 12 (16), 18769–18776. https://doi.org/10.1021/acsami.0c01202.

(12) Jang, W.; Kim, K.; Kim, B. G.; Nam, J.-S.; Jeon, I.; Wang, D. H. Prevention of Noise Current Generation in Tin-Based Lead-Free Perovskites for Highly Sensitive Photodetection. Adv. Funct. Mater. 2022, 32 (51), 2207713. https://doi.org/10.1002/adfm.202207713.

(13) Nam, J.-S.; Jang, W.; Han, J.; Kim, B. G.; Lim, J. H.; Kim, D.; Chung, I.; Wang, D. H.; Jeon, I. Enhanced Photodetection and Air Stability of Lead-Free Tin Perovskite Photodiodes via Germanium Incorporation and Organic Cation-Mediated Dimensionality Control. Adv. Funct. Mater. 2024, 34 (44), 2407299. https://doi.org/10.1002/adfm.202407299.

(14) Hussain, A. A. Constructing Caesium-Based Lead-Free Perovskite Photodetector Enabling Self-Powered Operation with Extended Spectral Response. ACS Appl. Mater. Interfaces 2020, 12 (41), 46317–46329. https://doi.org/10.1021/acsami.0c14083.

(15) Hussain, A. A.; Rana, A. K.; Ranjan, M. Air-Stable Lead-Free Hybrid Perovskite Employing Self-Powered Photodetection with an Electron/Hole-Conductor-Free Device Geometry. Nanoscale 2019, 11 (3), 1217–1227. https://doi.org/10.1039/C8NR08959K.

(16) López-Fernández, I.; Valli, D.; Wang, C.-Y.; Samanta, S.; Okamoto, T.; Huang, Y.-T.; Sun, K.; Liu, Y.; Chirvony, V. S.; Patra, A.; Zito, J.; De Trizio, L.; Gaur, D.; Sun, H.-T.; Xia, Z.; Li, X.; Zeng, H.; Mora-Seró, I.; Pradhan, N.; Martínez-Pastor, J. P.; Müller-Buschbaum,

P.; Biju, V.; Debnath, T.; Saliba, M.; Debroye, E.; Hoye, R. L. Z.; Infante, I.; Manna, L.; Polavarapu, L. Lead-Free Halide Perovskite Materials and Optoelectronic Devices: Progress and Prospective. Adv. Funct. Mater. 2024, 34 (6), 2307896. https://doi.org/10.1002/adfm.202307896.

(17) Li, Z.; Zhou, F.; Yao, H.; Ci, Z.; Yang, Z.; Jin, Z. Halide Perovskites for High-Performance X-Ray Detector. Mater. Today 2021, 48, 155–175. https://doi.org/10.1016/j.mattod.2021.01.028.

(18) Zhang, H.; Gao, Z.; Liang, R.; Zheng, X.; Geng, X.; Zhao, Y.; Xie, D.; Hong, J.; Tian, H.; Yang, Y.; Wang, X.; Ren, T.-L. X-Ray Detector Based on All-Inorganic Lead-Free Cs2AgBiBr6 Perovskite Single Crystal. IEEE Trans. Electron Devices 2019, 66 (5), 2224–2229. https://doi.org/10.1109/TED.2019.2903537.

(19) Pan, W.; Wu, H.; Luo, J.; Deng, Z.; Ge, C.; Chen, C.; Jiang, X.; Yin, W.-J.; Niu, G.; Zhu, L.; Yin, L.; Zhou, Y.; Xie, Q.; Ke, X.; Sui, M.; Tang, J. Cs2AgBiBr6 Single-Crystal X-Ray Detectors with a Low Detection Limit. Nat. Photonics 2017, 11 (11), 726–732. https://doi.org/10.1038/s41566-017-0012-4.

(20) Steele, J. A.; Pan, W.; Martin, C.; Keshavarz, M.; Debroye, E.; Yuan, H.; Banerjee, S.; Fron, E.; Jonckheere, D.; Kim, C. W.; Baekelant, W.; Niu, G.; Tang, J.; Vanacken, J.; Van der Auweraer, M.; Hofkens, J.; Roeffaers, M. B. J. Photophysical Pathways in Highly Sensitive Cs2AgBiBr6 Double-Perovskite Single-Crystal X-Ray Detectors. Adv. Mater. 2018, 30 (46), 1804450. https://doi.org/10.1002/adma.201804450.

(21) Yang, B.; Pan, W.; Wu, H.; Niu, G.; Yuan, J.-H.; Xue, K.-H.; Yin, L.; Du, X.; Miao, X.-S.; Yang, X.; Xie, Q.; Tang, J. Heteroepitaxial Passivation of Cs2AgBiBr6 Wafers with Suppressed Ionic Migration for X-Ray Imaging. Nat. Commun. 2019, 10 (1), 1989. https://doi.org/10.1038/s41467-019-09968-3.

(22) Yuan, W.; Niu, G.; Xian, Y.; Wu, H.; Wang, H.; Yin, H.; Liu, P.; Li, W.; Fan, J. In Situ Regulating the Order–Disorder Phase Transition in Cs2AgBiBr6 Single Crystal toward the Application in an X-Ray Detector. Adv. Funct. Mater. 2019, 29 (20), 1900234. https://doi.org/10.1002/adfm.201900234.

(23) Tailor, N. K.; Ghosh, J.; Afroz, M. A.; Bennett, S.; Chatterjee, M.; Sellin, P.; Satapathi, S. Self-Powered X-Ray Detection and Imaging Using Cs 2 AgBiCl 6 Lead-Free Double Perovskite Single Crystal. ACS Appl. Electron. Mater. 2022, 4 (9), 4530–4539. https://doi.org/10.1021/acsaelm.2c00752.
